# Monkeypox pandemic in Sudan, surveillance epidemiologic report, 2022

**DOI:** 10.1186/s12889-024-19058-9

**Published:** 2024-09-10

**Authors:** Ahmad Izzoddeen, Omer Elbadri, Mohamed Nageeb Abdalla, Mustafa Magbol, Muntasir Osman

**Affiliations:** 1 Field Epidemiology Training Program, FETP, FMoH, Khartoum, Sudan; 2 University of Gezira, Wad Medani, Sudan; 3 Faculty of Medicine, University of Al-Zaiem Al-Azhari, Khartoum, Sudan

**Keywords:** Mpox, Pandemic, Monkeypox, Surveillance

## Abstract

**Background:**

Mpox, is a zoonosis that is known to be endemic in several Central and West African countries. Recently, in 2022, it has emerged in Europe and United States, what raised the alarm to be declared in July 2022 as a public health event of international concern. This study aimed to give insight about the recent spread of mpox in Sudan, and documents the epidemiologic situation.

**Methods:**

Through a cross-sectional design, Sudan mpox data was extracted from the disease surveillance line-list at the national level at Sudan Federal Ministry of Health. the data was customized and then analyzed using Epi Info7 software. Analysis was done using frequencies and percentages and the results presented in tables and figures. Permission and ethical approval were obtained from the Health Emergency and Epidemic Control Directorate at the Federal Ministry of Health.

**Results:**

The outbreak of mpox was confirmed after testing of initial specimens outside Sudan with positivity rate of 72%. Later the cases continued to be reported based on the clinical diagnosis and standard case definition. Out of 375 reported cases, 54.4% were males, while 45.6% were females. The age of cases ranged from one month to 78 years with majority (41.1%) of the cases were children under 5 years of age. Regarding the reported symptoms, all cases had the characteristic skin rash and 74.1% of them had fever. Other symptoms included, headache (31.5%), sore throat (30.9%) and lymphadenopathy (26.1%). For occupation, 35.7% were preschool and 10.4% were school children, 9% of cases were prisoners. Around 22 (5.8%) reported contact history with a confirmed case, while (5.6%) of the cases were imported cases. Cases were reported from 17 states with 42 affected localities (districts) with an overall attack rate of 2.36/ 100,000. The highest number of cases was reported from Gadaref (45.3%), West Darfur (25.9%), Khartoum (13.3%) and north Darfur (3.5%). In Gadaref, 146 (85.8%) of the cases were from a refugees’ camp. Started in epi week 19, the outbreak peaked in week 38 and last in week 42.

**Conclusion:**

Mpox was confirmed in the new Sudan for the first time with cases reported in most of states. Although importation of the virus is hypothesized, internal hidden circulation is possible and more in-depth investigation is highly recommended. The higher rate of infection among preschool, school children and refugees, highlights the need to strengthen the prevention and control measures in schools and camps. More focus on the data completeness is required for better understanding of the disease and can be ensured by the surveillance directorate through training of staff and updating of reporting forms. Strengthening the lab capacity inside the country is a necessity to ensure testing of all the clinically diagnosed cases.

## Introduction

Monkeypox (recently named mpox) is a zoonosis endemic in several Central and West African countries, for which the animal reservoir has yet to be discovered [1]. Mpox is a double-stranded DNA virus known to be the most prevalent orthopoxvirus in humans [8]. On September, 1970, and during the eradication efforts of smallpox, the virus was isolated from a 9-month-old child suspected to have smallpox in the Democratic Republic of the Congo (DRC) [2]. Since then, mpox infections have occasionally occurred in 11 African countries in the followed decades [3]. Periodic outbreaks have led the virus to be endemic in west and central Africa [4] where most of the cases have occurred. Since 2016, the number of cases has started to increase even in those countries that have not reported cases of mpox for decades, in central Africa republic [18], the DRC (> 1000 reported per year), the Republic of Congo (88), Nigeria (> 80), Cameroon (80), Liberia [2], and Sierra Leone [1], reported cases respectively [5]. Mpox is transmitted to humans through close contact with an infected person or animal or with material contaminated with the virus. Patients with mpox typically experience a febrile prodrome 5–13 days after exposure (range = 4–17 days), which often includes lymphadenopathy, malaise, headache, and muscle aches; this prodrome might depend on the nature of exposure [6]. The prodrome is followed, 1–4 days later, by the onset of a characteristic deep-seated, vesicular or pustular skin rash with a centrifugal distribution; the lesions are well circumscribed and often umbilicate or become confluent, progressing over time to scabs [7]. Two clades of mpox virus were recognized: the West African and the Congo Basin. The more severe disease is caused by the second clade [3]. The case-fatality ratio for the West African clade of mpox is reported to be 1% and might be higher in immunocompromised persons [7].

Outbreaks were hypothesized as a consequence of general decline in population immunity to Orthopoxvirus due to the discontinuation of smallpox vaccination program 30 years ago, which provided protection against mpox also according to the World Health Organization (WHO) [8].

Despite the fact that it is known to be endemic in some parts of the African continent, the disease has been highlighted as one of the most important emerging diseases in the world [6] after mpox cases were identified in several countries in Europe in the period from January to June 2022, according to the World Health Organization [9]. One month later, the disease was declared by the WHO as a public health event of international concern (PHEIC) in July 2022 [6]. This pushed countries to consider the disease in their surveillance systems, particularly those with direct connections to endemic countries or those that have shared borders with them. Since the disease was not considered by the existing surveillance systems, its inclusion will be challenging for some countries. The surveillance system is already compromised and facing problems, including staff shortages, a lack of training, difficulties in communication, and a lack of supportive equipment and tools.

Since the disease started to become the focus of international health actors after the case was reported in Europe, Sudan began the effort to consider the disease under its surveillance. The health system in the country is already facing challenges due to the high burden of communicable and non-communicable diseases, further magnified by the poor infrastructure and gaps in data [10]. Keeping in mind that the disease is not endemic in Sudan, it is therefore not included in the country’s list of priority diseases for surveillance.

According to literature, Sudan reported mpox only one time in history, that was in October 2005, a child and his mother from southern Sudan [11]. The total reported cases at that time, as documented by Pierre Formenty et al., were 10 lab-confirmed and 9 probable cases with no reported deaths [12]. The source of infection was not clear, however the identified MPXV strain was found to be related to the Congo Basin clade [12, 13]. That was all occurred in Sudan before independence of South Sudan as a separate country in 2011, therefore it can be said that the new Sudan has never reported any mpox case before.

This report aimed to document the first mpox outbreak in Sudan after the independence of South Sudan. This provides insight about the pandemic in Sudan and the mpox epidemiologic situation, in addition to documentation and sharing of the country experience.

## Methodology

Mpox case definition, as adopted by FMOH, a suspected case of mpox is a person of any age presenting with unexplained acute rash and one or more of the following signs or symptoms; headache, acute onset of fever (> 38.5°C), myalgia, back pain, asthenia or lymphadenopathy.

A confirmed cases is a case meeting the cases definition and is laboratory confirmed for mpox virus by detection of unique sequences of viral DNA either by real-time polymerase chain reaction (PCR) and or sequencing. For tested cases only RT-PCR was used for confirmation. The cases were classified as imported if the person get the infection from, and spent the incubation period outside Sudan.

Due to unavailability of mpox testing in Sudan, the outbreak was confirmed through collecting specimens from a sample of suspected cases and sent to an outside laboratory, then cases continued to be reported based on the standard case definition and the clinical diagnosis.

Through a cross-sectional descriptive design, we conducted a retrospective epidemiologic analysis (time, place and person) for mpox cases reported to the national Surveillance and Information Directorate (SID) at Sudan Federal Ministry of Health (FMOH) from May 2022, to October 2022. Mpox data was extracted from the mpox national surveillance line-list where all reported cases details from all the reported states compiled into one Microsoft Excel sheet. The sheet was cleaned and customized, then imported to the Epi Info7 software for analysis. Analysis was done using frequencies and percentages and the results presented in tables and charts. Permission and ethical approval were obtained from the internal research committee at Health Emergency and Epidemic Control Directorate at the FMOH.

The attack rate was measured for each state by considering the population at risk in only the affected localities.

## Results

### Outbreak confirmation

To confirm the disease, 25 samples were collected and sent to Specialized Laboratories at the Robert Koch Institute in Germany. Cases were tested through Real-time polymerase chain reaction (RT-PCR) and MPXV was identified in 18 (72% positivity rate) and the outbreak was confirmed on 28th July 2022. Later, although specimens continued to be collected testing was difficult due to unavailability of the lab and the SID depends mainly on the clinical diagnosis.

### Person distribution

**Fig. 1:**
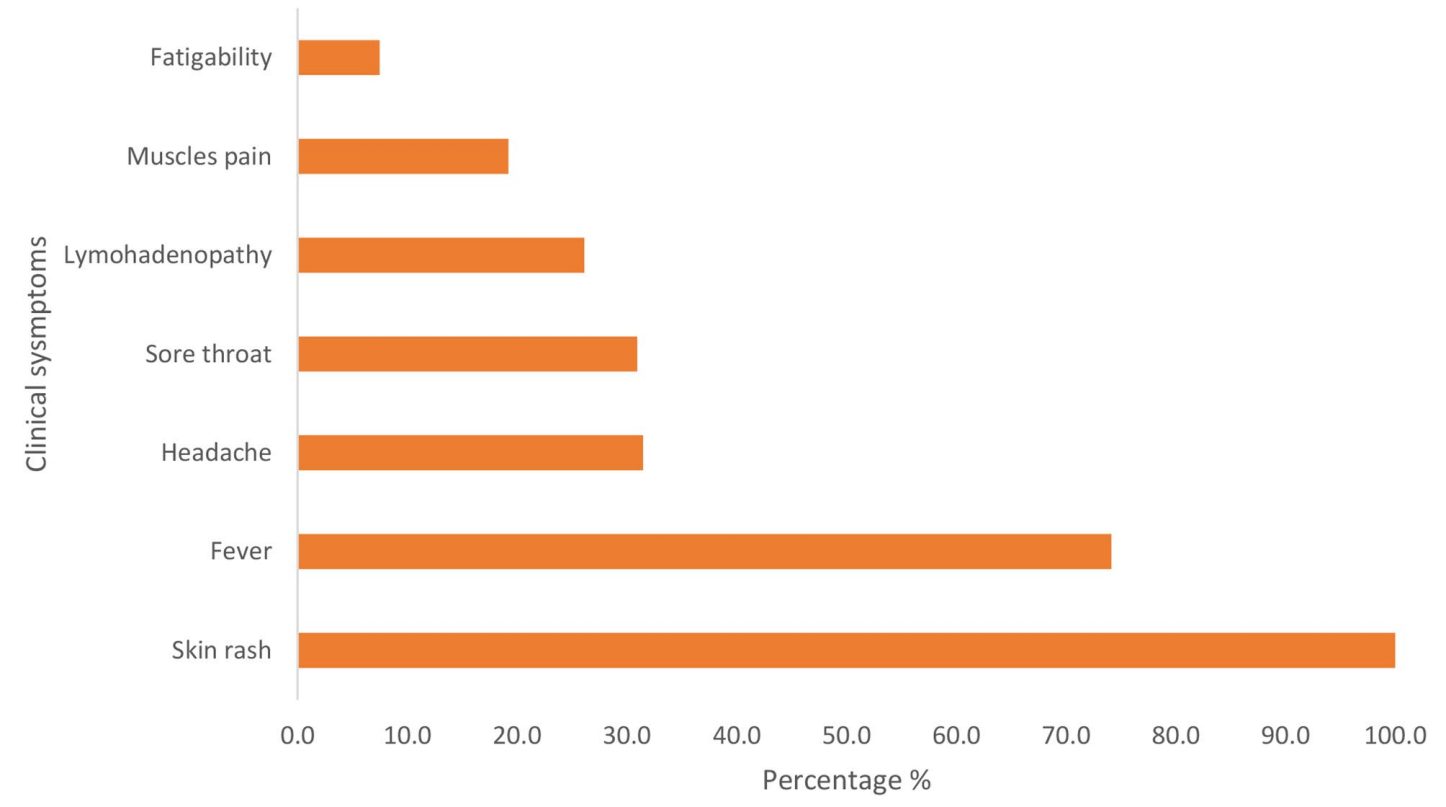
Distribution of clinical symptoms among mpox cases, Sudan, 2022 (*n* = 375)

This analysis covered 375 records of mpox cases extracted from the national surveillance line-list. Out of the total reported cases, 54.4% were males, while 45.6% were females (Table 1). The age of cases ranged from one month to 78 years with majority (41.1%) of the cases were children under 5 years of age (Table 1). Regarding the reported symptoms, all cases had skin rash and 74.1% of them had fever. Other symptoms included, Headache (31.5%), sore throat (30.9%) and lymphadenopathy (26.1%) (Table 2; Fig. 1). For occupation, 35.7% were preschool and 10.4% were school children, other patients’ occupations were, freelancing (4%), housewife (3.2%), prisoners (9%) and farmers (2.1%) (Table 1). Around 22 (5.8%) reported contact history with a confirmed case, while (5.6%) of the cases were imported cases. The imported cases were mainly from Saudi Arabia, Yemen, South Sudan, and Chad, these were detected by the Point of Entry (POE) surveillance (Table 2).

**Table 1:** Distribution of cases by gender, age, state and occupation, Sudan, 2022

Variable	Values	Frequency	Percentage
Sex		*n* = (375)	
	Females	171	45.60%
	Males	204	54.40%
Age group in years		*n* = (375)	
	0.0-4.9	154	41.1
	5-9.9	68	18.1
	10-14.9	34	9.1
	15-19.9	30	8.0
	20-24.9	17	4.5
	25-34.9	20	5.3
	40&more	52	13.9
Most affected states		*n* = 375	
	Gadaref	170	45.3%
	West Darfur	97	25.9%
	Khartoum	50	13.3%
	North Darfur	13	3.5%
	Central Darfur	9	2.4%
	Kassala	6	1.6%
	Other states	30	8%
Occupation		*n* = 232	
	Pre-school	134	35.7%
	School student	39	10.4%
	Freelancer	15	4.0%
	Housewife	12	3.2%
	Prisoner	9	2.4%
	Farmer	8	2.1%
	Not working	5	1.3%
	Government employee	5	1.3%
	Herdsman	3	0.8%
	Health worker	2	0.5%

**Table 2:** Distribution of Cases by clinical symptoms and contact history

Variable	Values	Frequency	Percentage
Symptoms		*n* = 375	
	Skin rash	375	100.0%
	Fever	278	74.1%
	Headache	118	31.5%
	Sore throat	116	30.9%
	Lymphadenopathy	98	26.1%
	Back pain	36	9.6%
	Muscle pain	36	9.6%
	Fatigue	28	7.5%
Travelling and contact			
	Contact	22	5.87%
	Imported case	21	5.60%
Country of importation			
	Saudi Arabia	16	4.53%
	Yemen	2	0.53%
	South Sudan	2	0.53%
	Chad	2	0.53%

### Place distribution

**Fig. 2:**
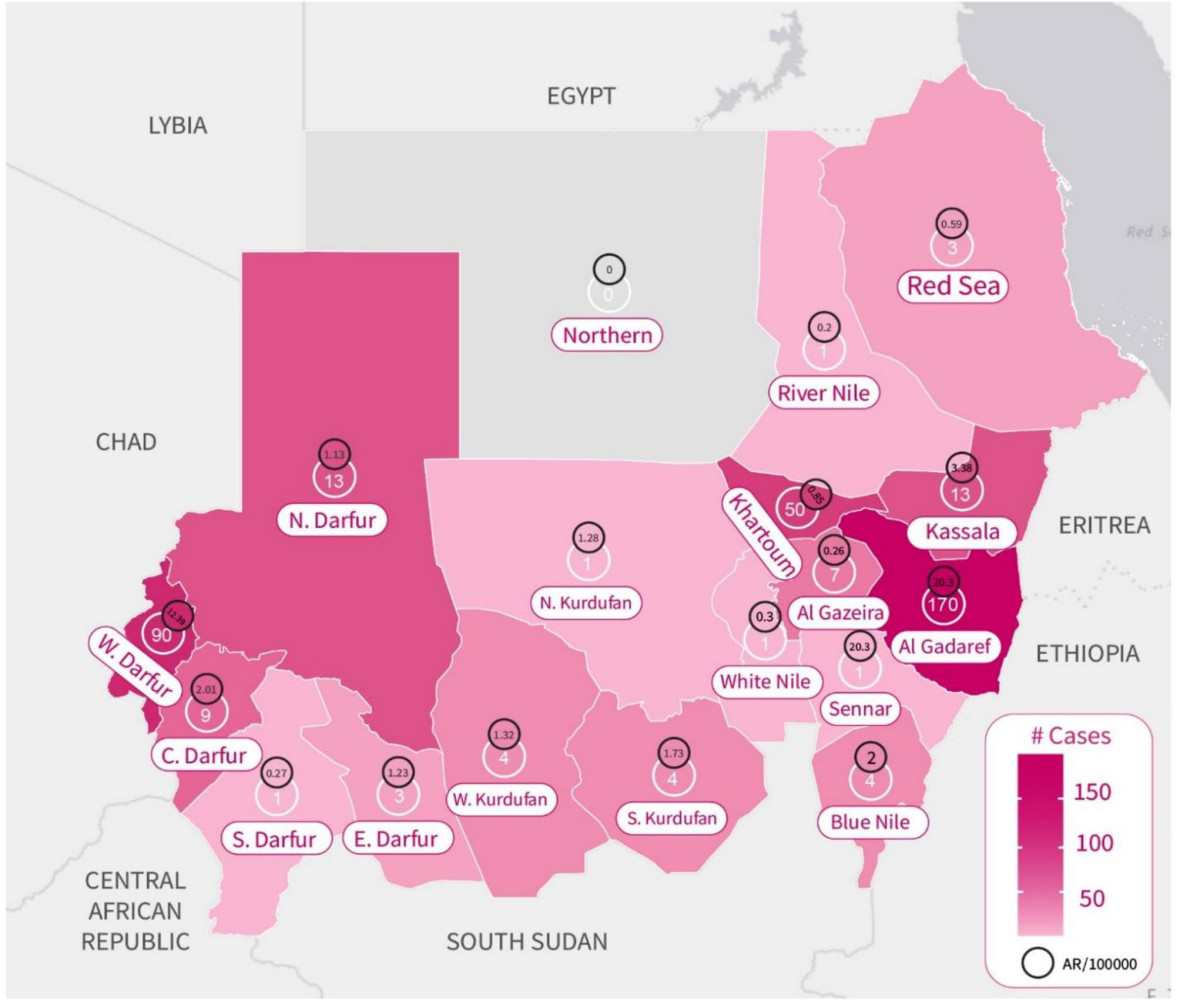
The map displays the cases load and the attach rates in the affected states, Sudan, 2022

Cases were reported from 17 states with 42 affected localities (districts). The total population at risk was 15,837,557 with an overall attack rate of 2.36/ 100,000. The highest number of cases was reported from Gadaref (45.3%), West Darfur (25.9%), Khartoum (13.3%) and north Darfur (3.5%) (Table 1; Fig. 2). In Gadaref state, 146 (85.8%) of the cases were from Tinaidba refugees camp in Mafaza locality. The case-load and the attack rates for al the affected states were represented in Fig. 2. The attck rate was highest in Gadaref (20.3/ 100,1000), followed by West Darfur (12.3/ 100,000), Kassala (3.3/ 100,000) and Central Darfur (2/100,000). Other states’ attack rates are demonstrated in Fig. 2.

### Time distribution of cases

**Fig. 3:**
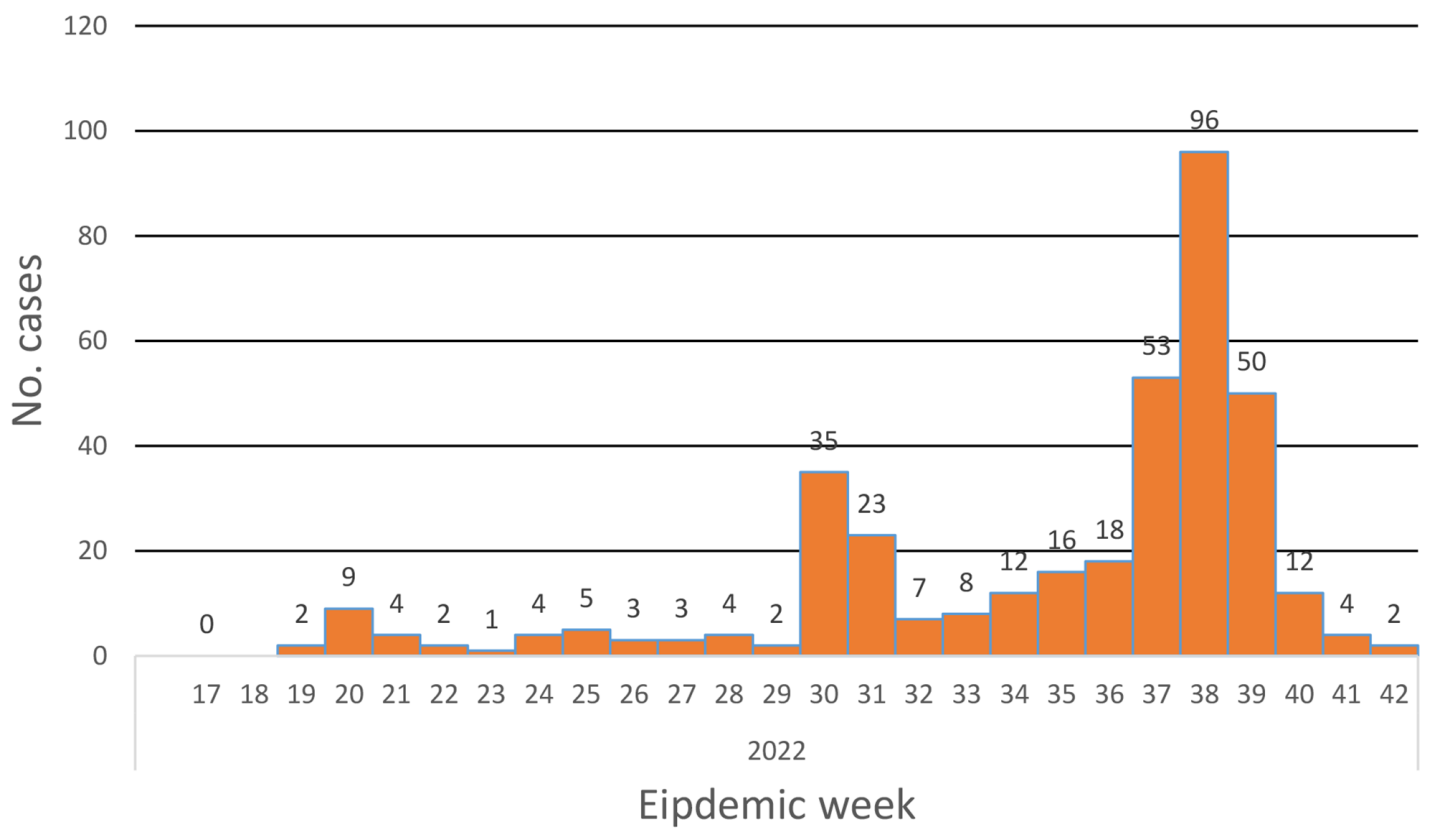
The epidemic curve of monkeypox epidemic in Sudan, 2022 (*n* = 375)

The first case was reported in 10 June 2022 (epi week 19), then the number of reported cases began to increase week by week to reach a peak of 101 reported cases in the epidemic week 38 level (Fig. 3). Those were mainly from Gedaref state, they were refugees in a refugees’ camp reported through the Early Warning Alert and Response Network (EWARN). Marked decline within 4 weeks was seen in the reported cases to reach only 2 cases in week 42, with 0 case reported after.

## Discussion

Although mpox was circulated before in southern Sudan (now the Republic of South Sudan), It has never been reported in other parts of the country and this is the first time it emerges. There was a strong theory that it was imported from another country, supported with that the first cases were from a border state with reported history of regular travelling across borders. However, the virus might also be already circulating inside the country and brought to the health authority focus after the declaration of the pandemic. More in-depth analysis and investigation was highly recommended to determine the origin of the infection. The reported cases showed slightly higher incidence among males compared to females. That looks the same as documented in other African countries [14], while the ratio is the same in north American countries [15]. Our study also reported increased rate of the diseases among pre-school and school children as nearly 60% of cases were under 10 years of age. The disease is well recognized to affect children compared to other age groups [14, 15]. In Sudan, generally skin diseases are prevalent and particularly among children aging 1 year to 18 year-old [16]. Children spread the infection between each other through playing and direct contact with siblings [17]. The hygiene plays a key role in prevention against skin diseases, inversely poor hygiene is a risk for contracting the diseases. That also might explain the higher number of cases reported from the refugees’ camp in Gadaref state, as poor hygiene is commonly linked to camps environments and is a continued challenge to skin diseases control [18, 19]. Skin infections is a well-known health challenge in Sudan, particularly in camps and closed gathering residences, like orphanages as reported in Darfur region [20]. The disease was reported from almost all the country, as cases were identified in 17 out of 18 Sudan states. The surveillance system in Sudan is mainly sentinel-based and recently event-based surveillance was also established [21]. More efforts are recommended to be put on the active surveillance, where cases are actively searched and investigated within the communities by the rapid response teams. This will have better outcome regarding detection of cases and identification of the potential infection sources. The clinical features ranged from rash with fever to being accompanied by headache with/ without lymphadenopathy. These were the major symptoms that the surveillance system depended on in detecting cases, the case definition was adapted from the WHO case definition for monkeypox [7, 22].

Occupation information was not obtained for considerable number of patients, this needs careful review by the surveillance directorate, as occupation is one of the important exposure variables and helps understanding the disease spread and source of infection. Other very important variables that are key in identifying the source of infection and risk factors are the contact history and the travel history. Only 5.8% reported contact history with a confirmed case, and 5.6% of the cases were imported cases.

The disease was newly emerging and is not part of the priority notifiable diseases. This might serve as a challenge for surveillance workers to understand the diseases and collect the appropriate data, and also challenged the data completeness. Provision of training to frontline surveillance staff at health facilities and localities and provision of structured data collection forms will all ensure improved data completeness and better analysis.

The limited laboratory testing capacity in Sudan stand against testing all cases, as only the initial cases were tested outside the country, what led the health authorities to depend on clinical diagnosis through the standard cases definition. That highlighted the need for improving the national lab readiness by training of the staff and ensuring the availability of laboratory supplies.

## Conclusion

Mpox was confirmed in the new Sudan for the first time with cases reported in most of states. Although importation of the virus is hypothesized, internal hidden circulation is possible and more in-depth investigation is highly recommended. The higher rate of infection among preschool, school children and refugees, highlights the need to strengthen the prevention and control measures in schools and camps. More focus on the data completeness is required for better understanding of the diseases and can be ensured by the surveillance directorate through training of staff and development of reporting forms. Strengthening the lab capacity inside the country is a necessity to ensure testing of all the clinically diagnosed cases.

### Limitations

Generally, there is a limited published data on the mpox leaving a large gap on the country history and profile with the disease, what in turn stands against enriching the discussion to improve the insight. Moreover, the data provided and available in FMOH is limited and not permitted advance analysis for more understanding of the risk of infection and potential source of infection. Data on the circulating virus serotype was not provided by the lab.

## Data Availability

the datasets analyzed during this current study are not publicly available due to policy and restriction of Federal Ministry of Health, Surveillance Department, Sudan. However, are available from the corresponding author on reasonable request.
