# Correction: Monkeypox pandemic in Sudan, surveillance epidemiologic report, 2022

**DOI:** 10.1186/s12889-024-20385-0

**Published:** 2025-03-06

**Authors:** Ahmad Izzoddeen, Omer Elbadri, Mohamed Nageeb Abdalla, Mustafa Magbol, Muntasir Osman

**Affiliations:** 1 Field Epidemiology Training Program, FETP, FMoH, Khartoum, Sudan; 2 University of Gezira, Wad Medani, Sudan; 3 Faculty of Medicine, University of Al-Zaiem Al-Azhari, Khartoum, Sudan

**Correction: BMC Public Health (2024) 24:2457**

10.1186/s12889-024-19058-9

The original publication of this article contained a typo in the abstract, the incorrect and correct information is listed in this correction article. The original article has been updated.

Incorrect

June 2024

Correct

July 2022

